# Geographical distributions of African malaria vector sibling species and evidence for insecticide resistance

**DOI:** 10.1186/s12936-017-1734-y

**Published:** 2017-02-20

**Authors:** Antoinette Wiebe, Joshua Longbottom, Katherine Gleave, Freya M. Shearer, Marianne E. Sinka, N. Claire Massey, Ewan Cameron, Samir Bhatt, Peter W. Gething, Janet Hemingway, David L. Smith, Michael Coleman, Catherine L. Moyes

**Affiliations:** 1 Malaria Atlas Project, Oxford Big Data Institute, Li Ka Shing Centre for Health Information and Discovery, University of Oxford, Oxford, OX3 7BN UK; 2 Department of Vector Biology, Liverpool School of Tropical Medicine, Pembroke Place, Liverpool, L3 5QA UK; 3 Oxford Long Term Ecology Laboratory, Department of Zoology, University of Oxford, Oxford, OX1 3PS UK; 4 Department of Infectious Disease Epidemiology, Imperial College, St Mary’s Hospital, London, W2 1NY UK; 5 Institute for Health Metrics and Evaluation, University of Washington, Seattle, WA 98121 USA

**Keywords:** Species distribution model, Maps, Susceptibility bioassays

## Abstract

**Background:**

Many of the mosquito species responsible for malaria transmission belong to a sibling complex; a taxonomic group of morphologically identical, closely related species. Sibling species often differ in several important factors that have the potential to impact malaria control, including their geographical distribution, resistance to insecticides, biting and resting locations, and host preference. The aim of this study was to define the geographical distributions of dominant malaria vector sibling species in Africa so these distributions can be coupled with data on key factors such as insecticide resistance to aid more focussed, species-selective vector control.

**Results:**

Within the *Anopheles gambiae* species complex and the *Anopheles funestus* subgroup, predicted geographical distributions for *Anopheles coluzzii*, *An. gambiae* (as now defined) and *An. funestus* (distinct from the subgroup) have been produced for the first time. Improved predicted geographical distributions for *Anopheles arabiensis*, *Anopheles melas* and *Anopheles merus* have been generated based on records that were confirmed using molecular identification methods and a model that addresses issues of sampling bias and past changes to the environment. The data available for insecticide resistance has been evaluated and differences between sibling species are apparent although further analysis is required to elucidate trends in resistance.

**Conclusions:**

Sibling species display important variability in their geographical distributions and the most important malaria vector sibling species in Africa have been mapped here for the first time. This will allow geographical occurrence data to be coupled with species-specific data on important factors for vector control including insecticide resistance. Species-specific data on insecticide resistance is available for the most important malaria vectors in Africa, namely *An. arabiensis*, *An. coluzzii*, *An. gambiae* and *An. funestus*. Future work to combine these data with the geographical distributions mapped here will allow more focussed and resource-efficient vector control and provide information to greatly improve and inform existing malaria transmission models.

**Electronic supplementary material:**

The online version of this article (doi:10.1186/s12936-017-1734-y) contains supplementary material, which is available to authorized users.

## Background

Over 100 anopheline mosquito species can transmit human malaria parasites but there are important differences among these species that influence their role in malaria transmission. Many of these species belong to a sibling complex; a complex is a taxonomic group of morphologically identical, closely related species. In the past, sibling species have been hard to distinguish and complexes have often been treated as a single entity despite important differences among sibling species. In Africa, *Anopheles arabiensis*, *Anopheles coluzzii* and *Anopheles gambiae* from the Gambiae complex and *Anopheles funestus* from the Funestus subgroup are undoubtedly the most important vectors transmitting both *Plasmodium falciparum* and *Plasmodium vivax* parasites to humans [1–3]. Within the Gambiae complex, *Anopheles melas* and *Anopheles merus* are also considered dominant vectors (“dominant” is defined as a vector species that has been identified as the main, dominant or important vector in at least one region) whereas there is no strong evidence that other species from this complex play any role in malaria transmission [4, 5].

In addition to differences in the vector status of each species, sibling species also have important differences in their geographical distributions. Previous studies that estimated the geographical distributions of the dominant malaria vectors were hampered by low volumes of data for individual sibling species and had to choose between mapping complexes or incorporating species records that had been determined on the basis of morphology alone and were therefore potentially misidentified [6]. Furthermore, insecticide resistance in vector species currently threatens the efficacy of vector control [7], making this a critical factor that needs to be understood within each vector species. In the past, many studies that used susceptibility assays to measure prevalence of resistance in vector populations did not fully identify sibling species. Thus, the mortality values obtained related to the species complex as a whole and potentially important differences among sibling species were not identified.

In recent years, the importance of species identification alongside the availability of accurate molecular identification methods has increased the number of studies reporting reliably identified sibling species. The aim of this study was to use the increasing availability of sibling species records, and an improved species distribution model, to define the geographical distributions of individual vector species within the Gambiae complex and Funestus subgroup in Africa. The available evidence for insecticide resistance was then examined in these species to assess the feasibility of combining insecticide resistance data with the geographical distributions generated. The distributions of *An. gambiae* and *An. coluzzii* are modelled separately for the first time and *An. funestus* is modelled for the first time as the type species distinct from other members of the subgroup.

## Methods

### Summary of species distribution map generation

Records of sibling species occurrence, where species were identified using molecular methods, were retrieved from the published literature (from both resistance and behavioural studies) and from unpublished sources to compile a set of presence records for each species. A larger dataset, including all *Anopheles* surveys in the region, was used as a background dataset that captured sampling bias in the presence records. Both datasets informed a species distribution model that identified the combinations of environmental variables that best distinguished areas supporting species presence from the range of environments sampled. This model was then used to estimate the relative probability of species presence at all locations within the species range.

### Species background and presence data

Data from two previously collated and publicly available databases of dominant malaria vector species occurrence and bionomics [8, 9] were combined with a new database of insecticide resistance records (described below) and duplicate records were removed. Searches of the more recent literature were conducted from the dates that the earlier searches finished (2009, 2013 and 2015 respectively) to 29 September 2016, to fill any gaps in the dataset. The new searches used the Web of Sciences bibliography and the search terms “[species name]” and “[country name]”. New records of occurrence that matched the inclusion criteria were extracted and added to the composite database. Only studies that provided the location and time of collection, and gave details of the identification method(s) used, were included.

Geographical coordinates for the collection locations were converted to decimal degrees. For sites where no coordinates were given, coordinates were assigned using the site name and contextual information, such as the district or distance to a major city, using online gazetteers including GeoNames, Google Maps, and OpenStreetMap. All coordinates provided by the source or generated as part of this project were checked to ensure that they matched the sampling design described, fell on land and fell in the correct country, using the geographical information software ArcMAP. If collection dates were missing for a data point, the year of collection was assumed to be two years before the article publication year based on the trend seen for data with a known collection date, for the purposes of this study. For each species, the full species occurrence dataset was classified into (1) studies that used molecular identification methods capable of detecting that species, and (2) studies that would not have detected that species using molecular methods.

To generate a presence dataset for each species, all records that used appropriate molecular identification methods and recorded presence of the species were extracted from the dataset described above. For all studies that identified the species formerly known as *An. gambiae* (*An. gambiae*/*An. coluzzii* or M/S forms combined) using molecular methods but did not identify the M and S forms, occurrences were labelled “*An. gambiae* (old)” to distinguish them from the newly defined *An. gambiae* (formerly *An. gambiae* S form). Records of “*An. gambiae* (old)” outside the *An. coluzzii* range plus a 300 km buffer were designated *An. gambiae* and records inside the overlapping *An. gambiae* and *An. coluzzii* ranges, plus buffer, were discarded.

It is not possible to test empirically whether spatial clustering in the presence data is due to habitat suitability or spatial bias in sampling effort, so this was accounted for a priori through the selection of background data with the same spatial bias in sampling effort [10]. The full mosquito occurrence dataset was used as a source of background data that captures the sampling bias in the environments surveyed for *Anopheles* species. Most of the species modelled have ranges that border on desert areas where *Anopheles* are known to be absent but no surveys are performed, so 210 pseudo-absences were generated by randomly selecting locations within the desert biome defined by the United Nations Environment Programme [11].

### Summary of available insecticide resistance data for sibling species

A literature search was performed in the Web of Science bibliographic database using the search terms “insecticide resistance” and “anopheles”. Articles of potential interest were identified and their abstracts were scanned to identify studies that had performed a bioassay on field-collected mosquitoes (up to the F1 generation). Full texts were obtained for these articles and data extracted for all bioassays that had used an insecticide from one of the four major neurotoxic classes: carbamates, organochlorines, organophosphates and pyrethroids. Unpublished data were also requested from authors of the published articles and groups working on insecticide resistance. Data fields extracted from both published and unpublished sources covered: species; collection dates; collection location; method(s) of capture; method(s) of identification; insecticide; bioassay protocol; percent mortality; and source citation(s). Data from all bioassays identified were extracted and any deviations from a standard published protocol (for example non-standard exposure times) were noted. Records that did not identify sibling species using molecular methods were discarded for the purposes of the current study. Summary statistics were calculated, based on the mortalities obtained for samples where >95% of the mosquitoes were identified as a single species, to give an indication of bioassay data availability and variation among sibling species.

### Species ranges

In order to model the geographical distribution within each species range, previously defined ranges for *An. arabiensis*, *An. funestus*, *An. melas* and *An. merus* [6, 12] were used to limit the extent of the model outputs. These ranges were compared to the presence dataset for each species described above and if confirmed records of the species were found outside the previously defined range, the range was extended to encompass the new location(s). For *An. coluzzii* the presence dataset and a previous map showing records of the M form of *An. gambiae* [13] were used to define its range. One record of a single *An. coluzzii* mosquito in Zimbabwe shown on the previous map was discarded after a thorough search of the literature found no other record of this species within over 500 km of this location since the original record was published. Individual species ranges and presence points were combined to generate ranges for the Gambiae complex, Funestus subgroup and Funestus group. A 300 km buffer was added to each species range to reflect uncertainty in the exact ranges of these mosquitoes.

### Environmental data

The modelling approach used here relies on the relationship between species occurrence and combinations of environmental covariates. Covariate values were extracted from an existing set of spatial data layers for environmental covariates believed to be of importance to mosquito occurrence and malaria transmission [14]. Full details are provided in Additional file 1. Briefly, data layers at a 5 × 5 km resolution were included for land surface temperature, seasonality in temperature, measures of wetness/greenness, seasonality in wetness/greenness, elevation, proportional cover of 14 land classes, and human population density.

### Species distribution model

Each species was modelled separately using the same species distribution model. The approach used was a boosted regression trees method that combines both regression trees (which build a set of decision rules on the predictor variables by portioning the data into successively smaller groups with binary splits) and boosting (which selects sets of trees that minimise the loss function) to best capture the variables that define the distribution of the input data [15–17]. The boosted regression trees methods has been used in previous malaria vector studies [6] and has recently been updated to use background data that characterises sampling bias in the presence records, and to include changes in land cover over time [18, 19]. For each model, the presence records for that species and the background data points located within the range of that species, excluding survey records found in the presence dataset, were used. The background data were classified into (1) records from studies that used methods that would have identified the sibling species being modelled (had that species been present in the sample collected), and (2) all other records. Data points linked to a random 10% of locations from both the presence and background datasets were withheld for use in the model validation. Together the remaining presence and background data formed the model training dataset.

The boosted regression trees method requires both presence and absence data, or background data can be used when true absence data is not available. Mosquito occurrence datasets are subject to spatial bias and if unaccounted for this survey bias can translate into environmental bias in the fitted model. The background data used in the study reflected the same survey bias found in the presence data so the model could identify suitable environments for the species within the sampled space, rather than just areas that are more heavily sampled. This approach does not eliminate sampling bias issues entirely but improved model performance has been demonstrated [10]. The model was updated further in order to weight the background data so that records from surveys using molecular methods that would have identified the species being modelled (had that species been present in the sample collected) received twice the weight of other background data points. Presence and background data from 2001 to 2012 were linked to covariate values for the relevant year in order to improve the predictions where possible. For all records prior to 2001, covariate values for 2001 were used, and for any data collected after 2012, covariate values for 2012 were used. Model predictions were made to the most contemporary covariate data available.

For each species, 200 submodels were then fitted trained to a bootstrap of the presence/background dataset. Each submodel generated a predicted value for the relative probability of species occurrence at every 5 km × 5 km pixel and together the ensemble of submodels generated a distribution of predicted values for every pixel. Mean values together with 0.025 and 0.975 quantile values were then derived from the distribution of predictions at every 5 × 5 km pixel.

### Model validation

Withheld data (the test data) from each presence and background dataset were used to validate each mean map generated. The area under the receiver operator curve (AUC) was calculated to assess the mean map’s ability to distinguish species presence points from background points that are representative of the locations surveyed for *Anopheles* vectors, whilst marginalising the arbitrary choice of a classification threshold [20]. An AUC of 0 means the model ranked all sites the wrong way round, 0.5 means the model was no better than random, and an AUC of 1 means it made a perfect prediction. The same test presence and background datasets were used to calculate the AUC value for previously published maps of these species to allow us to compare model performance.

## Results

### Compiled species occurrence database

The data volumes available for each species are given in Table 1, the presence and background data that went into the training and test datasets are provided in Additional file 2, and maps showing the distributions of these datasets are provided in Additional file 3.

**Table 1:** The volumes of data collated

Species	Number of presence points	Number of background points	
		Class 1	Class 2
*An. arabiensis*	2106 (505)	1066	784
*An. coluzzii*	1086 (172)	385	762
*An. funestus*	720 (172)	50	2991
*An. gambiae*	1703 (420)	1070	1058
*An. merus*	111 (71)	447	0
*An. melas*	178 (58)	1021	3

The total number of presence points for each species is provided and the subtotal that fell outside the time range for which the annual covariate data is given in parentheses. Background data points are split into those that used molecular methods that would have identified the species modelled (class 1) and those that did not (class 2)

### Geographical distributions of the sibling species

**Fig. 1:**
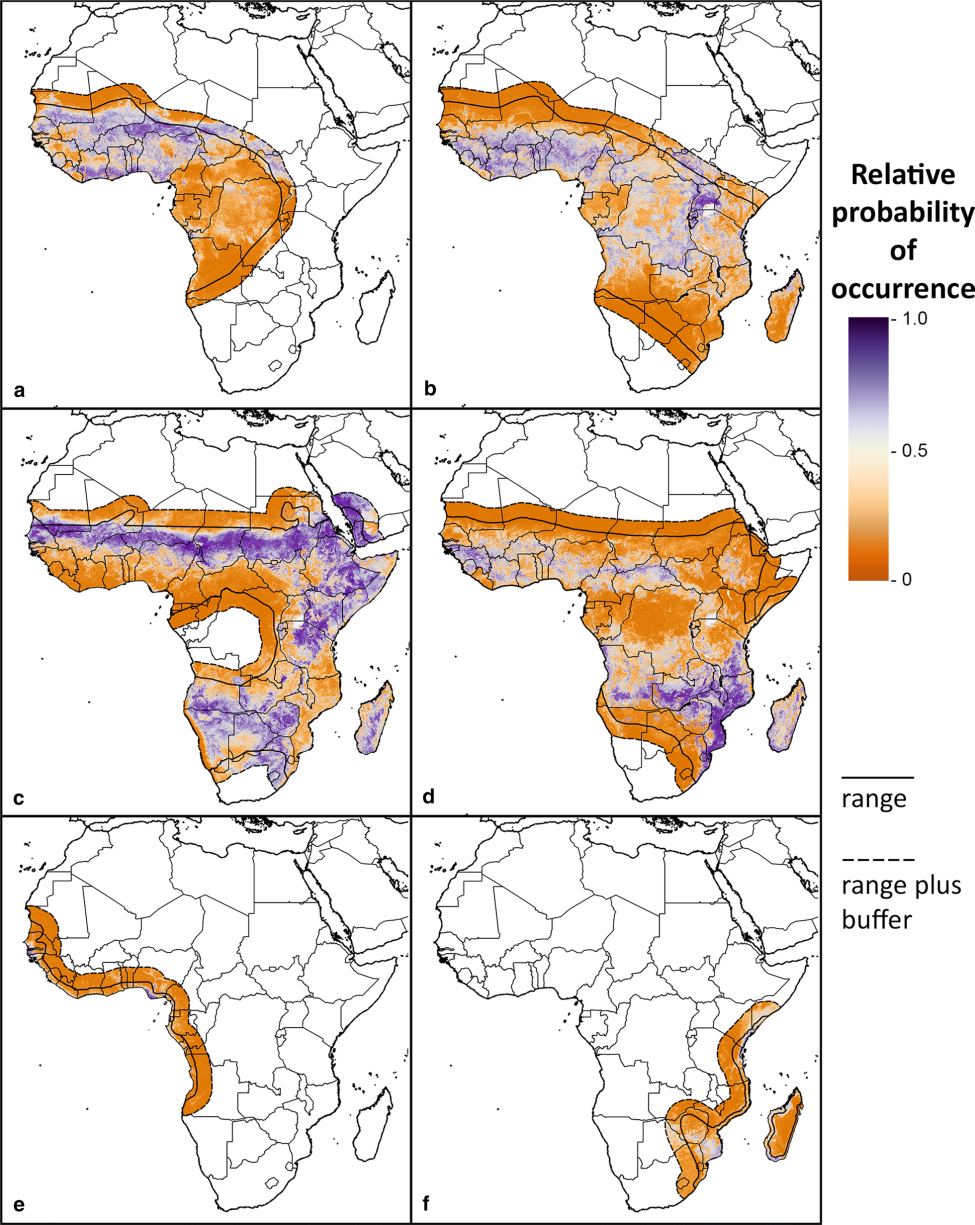
Predictive maps for occurrence of sibling species. The relative probability of occurrence for each species is shown within its range plus a 300 km buffer. **a** *An. coluzzii*. **b** *An. gambiae* **c** *An. arabiensis*. **d** *An. funestus.* **e** *An. melas*. **f** *An. merus*

The mean estimated relative probability of occurrence for each modelled sibling species is shown in a set of predictive maps (Fig. 1a–f). In addition, predictive maps for the Gambiae complex as a whole, Funestus subgroup and Funestus group are provided in Additional file 4.

The AUC values for the Gambiae complex model outputs were 0.870 for *An. arabiensis*, 0.783 for *An. coluzzii*, 0.778 for *An. gambiae*, 0.866 for *An. melas*, and 0.804 for *An. merus*. These values are consistently higher than those obtained for the previously published maps (available for *An. arabiensis*, *An. melas* and *An. merus* only) as shown in Additional file 5. The AUC values for the modelled *An. funestus* map was 0.824, for the Funestus subgroup it was 0.806 and for the Funestus group it was 0.796. The AUC value for the Funestus subgroup was higher than the value obtained for the previously published map (Additional file 5). Maps showing uncertainty in these predictions, in the form of the range from the 2.5th to the 97.5th centile, are provided in Additional file 5. The GeoTIFF files containing the mean, median and quantile predictions for every 5 × 5 km pixel are provided in Additional files 6, 7, 8, 9, 10, 11, 12, 13, 14.

The environmental variables that proved to be the top predictors in each species model are also provided in Additional file 5. For members of the Gambiae complex the top two predictors were always related to temperature and/or wetness, or elevation which is closely correlated with both. For *An. funestus*, land cover types were also strong predictors.

For the most part, there are few contiguous areas of high relative probability of occurrence that cross from the known range of a species into the 300 km outside this range, indicating biogeographical barriers are limiting the species ranges (Fig. 1). For *An. coluzzii*, however, an area of high relative probability of occurrence, or environmental suitability, can be seen running from within its range in Chad out to the northwest. A similar area in South Sudan can be seen for *An. gambiae*. This could indicate areas suitable for future expansion of the ranges of these species, or it could reflect uncertainty about the current species ranges in this part of Africa. The same pattern can be seen at the southern end of the *An. arabiensis* range where this species may be restricted by factors that have not been captured in this model.

### Insecticide resistance in sibling species

Bioassay results from samples where more than 95% of the mosquitoes were identified as belonging to a single species were included in the final insecticide resistance dataset, providing 2437 records. The results for each insecticide class and species are given in Additional file 15 and the results for pyrethroid resistance in members of the Gambiae complex are shown in Table 2. A further 1156 records provided mortality records for mixed samples of identified sibling species within the Gambiae complex and 424 records came from studies that used molecular methods to identify species but did not provide the species composition of the samples.

**Table 2:** Available data on pyrethroid resistance for sibling species of the Gambiae complex

Year range	No. records	Mortality (%)	Countries
*An. arabiensis*			
Up to 2000 (first year = 1996)	3	Min 100	South Africa
		Max 100	
		*Mean 100*	
2001–2005	67	Min 75	Cameroon, Kenya, Madagascar, Mozambique, Nigeria, South Africa, Sudan, Tanzania
		Max 100	
		*Mean 95.6*	
2006–2010	218	Min 0	Burkina Faso, Cameroon, Chad, Ethiopia, Kenya, Mozambique, Senegal, Sudan, Tanzania, The Gambia, Uganda, Zambia, Zimbabwe
		Max 100	
		*Mean 79.3*	
2011–2015	161	Min 9	Ethiopia, Kenya, Malawi, Mali, South Africa, Senegal, Sudan, Tanzania, Uganda
		Max 100	
		*Mean 74.7*	
*An. coluzzii*			
2001–2005	38	Min 19	Benin, Cameroon, Nigeria
		Max 100	
		*Mean 89.7*	
2006–2010	144	Min 0.9	Benin, Burkina Faso, Cameroon, Côte d’Ivoire, Ghana, Mali, Nigeria
		Max 100	
		*Mean 73.2*	
2011–2015	44	Min 1	Benin, Burkina Faso, Cameroon, Côte d’Ivoire, Mali, Mozambique, Liberia
		Max 100	
		*Mean 60.6*	
*An. gambiae*			
Up to 2000 (first year = 1999)	10	Min 100	Zambia
		Max 100	
		*Mean 100*	
2001–2005	55	Min 27	Angola, Burundi, Cameroon, Equatorial Guinea, Mozambique, Nigeria, Uganda
		Max 100	
		*Mean 88.82*	
2006–2010	139	Min 0	Benin, Burkina Faso, Burundi, Cameroon, Congo, Ghana, Guinea, Kenya, Malawi, Mozambique, Uganda, Zambia
		Max 100	
		*Mean 75.2*	
2011–2015	48	Min 0	Cameroon, DRC, Ghana, Kenya, Mali, Tanzania, Uganda
		Max 100	
		*Mean 48.7*	
*An. melas*			
All years (2005)	4	Min 100	Cameroon
		Max 100	
		*Mean 100*	
*An. quadriannulatus*			
All years (2002)	1	Min 100	South Africa
		Max 100	
		*Mean 100*	

A record is defined as a mortality value for a single mosquito population sampled at a specified time and place by a unique study. For species with <10 bioassay records, records for all years were aggregated and the year range is noted in parentheses. For species with >10 bioassay records, the data was divided into three year ranges and the year of the first record is given in parentheses. The mean (given in italics), minimum (min) and maximum (max) mortality values across all records for that time period are given together with a list of the countries where the field collections were taken

When the criterion of >95% of the mosquitoes confirmed as a single sibling species is applied, it is apparent that since the year 2000 over 100 bioassay results from multiple countries have been made available for each of the following: pyrethroid resistance in *An. arabiensis*, *An. coluzzii*, *An. gambiae* and *An. funestus*; carbamate resistance in *An. arabiensis* and *An. coluzzii*; organochlorine resistance in *An. arabiensis* and *An. gambiae*; and organophosphate resistance in *An. arabiensis*. Far fewer data points are available for the years up to 2000 and the first results for sibling species start in the late 1990s. For other African vector species, available data are limited or were not found at all. The search included malaria vector species outside the Gambiae complex and Funestus group, but no available data were found for *An. coustani* (*An. coustani* data are available but not confirmed using molecular identification methods), *An. moucheti*, species of the Nili complex, or *An. pharoensis*.

Consistent susceptibility to organophosphates was found for *An. funestus* across all 11 countries sampled from 1999 to the current time, and was also seen in the small number of results for other members of this group. Within the Gambiae complex, differences are apparent among sibling species in terms of their resistance to each of the four major classes of insecticide (Table 2; Additional file 14). Caution is needed, however, when identifying apparent variation or trends in the insecticide resistance data. For example, the summary data in Table 2 appears to show that resistance to pyrethroids has consistently increased over time within members of the Gambiae complex but Table 2 also shows that there is substantial bias in the locations sampled and in the times sampled. These biases are likely also to be present at finer spatial and temporal scales, and need to be incorporated in any analysis of the patterns of resistance in sibling species. Further, the values shown are derived from bioassays that used a range of protocols and these differences need to be captured and included in the data in order to perform a robust analysis of the dataset.

## Discussion

This study provides full modelled geographical distributions for *An. coluzzii* and *An. gambiae* (as now defined) for the first time and clear differences can be seen between these two sibling species, formerly considered a single species. Estimates for the distributions of *An. arabiensis*, *An. melas* and *An. merus* (also within the Gambiae complex) are provided based on improved methods and updated data, resulting in notably better model performance than seen with a previous mapping study [6]. The geographical distribution of *An. arabiensis* has also been modelled in recent years by an independent group [21]. Their aim was to extrapolate into the future when environmental conditions not currently in existence may occur so they selected a low bias bootstrap aggregation for one class data (LOBAG-OC) model. The data output by their model were not released so a quantitative comparison is not possible but a visual comparison shows broader habitat suitability in the earlier modelled map compared to the current study. The AUC value generated by that study was marginally lower than the value generated here (0.77 compared to 0.78, although caution is needed because the data used to generate these values differed), the data volumes were much lower, and the data for each environmental variable used in the earlier model was a single average over a long time period (1950–2000), meaning the current map is based on a more robust approach.

Although the full geographical distributions of *An. coluzzii* and *An. gambiae* have not been modelled previously, a recent study modelled the probability of *An. coluzzii* presence relative to the probability of *An. gambiae* presence in their sympatric range [22]. It is difficult to compare (1) an analysis of the relative occurrence of two species with (2) two independent species maps, but the results presented here are consistent with the predictions made by the earlier study. Both show *An. coluzzii* extending further north and closer to the coast than *An. gambiae* within west Africa. An interesting extension of the current work would be to characterize the locations where both species are sympatric while maintaining reproductive isolation.

A modelled distribution for *An. funestus* (distinct from the subgroup) has also been generated here for the first time. Only range maps were previously available for this species but the geographical distribution of the Funestus subgroup has been modelled previously [6]. The geographical distribution for the Funestus subgroup generated by the current study, using the same methods as the current *An. funestus* map, showed better model performance than the earlier work.

It is well established that *Anopheles* species are strongly influenced by temperature and humidity or wetness [23–25], and previous studies have found differences in the relationships between these variables and individual sibling species [26–32]. The study presented here used a model that provides strong predictive power to generate robust species distributions but it cannot elucidate relationships with individual environmental variables. It was clear, however, that the variables with the strongest influence on each model were wetness and temperature or factors strongly correlated with these two variables, namely elevation and a vegetation index, and the exact set of top predictors varied among each sibling species. Relationships between species and vegetation type have also been found by previous studies [33, 34] and pollution is known to play a role [22]. Fourteen land classes were included in this work and some of these were important predictors as was human population density, which is linked to pollution, but caution is needed when interpreting the ranking of covariate influence by the model. The modelling framework used here is not an appropriate tool to confirm the specific relationships with environmental variables found by detailed field studies and the maps also need to be viewed in the context of the scale used. The aim here was to produce continent-wide maps at a 5 × 5 km resolution and the land cover data used by the model were expressed as the proportion of square kilometres within each 5 × 5 km assigned to a particular land cover type. All other environmental and socioeconomic variables were provided to the model at 5 × 5 km resolution. This approach does not capture microscale variation that may be important to these species locally. At the other end of the spectrum, however, for vector and malaria control plans devised at a national or subnational level, the data from the maps presented here can be aggregated to provide information for the areas used for planning.

The temporal resolution of the data used to generate these maps was annual and thus seasonal variation is not captured here and the maps presented provide the relative probability of a species occurring at each location during at least one time of the year. Strong seasonal fluctuations in mosquito abundance occur, particularly in West Africa, and these differ among species [35–38]. If the dataset used here incorporated a systematic bias towards collection times that would miss particular species then this could impact the maps generated, however, the collated data provide good coverage for each species within areas with strong seasonal patterns as well as regions with smaller fluctuations (Additional file 3).

The geographical distribution of a species is not sufficient information alone to inform vector control programmes and these distributions need to be used in combination with data on the key attributes of each species. Use of indoor insecticide-based control measures has resulted in important reductions in vector populations and malaria prevalence [39, 40] but at the same time the relative abundance of individual malaria vectors has changed [41–44] leading to greater importance placed on mosquitoes that bite or rest outdoors or have less restricted feeding preferences [8, 45, 46]. Also critically important is resistance to the major insecticide classes and data is essential to provide evidence for insecticide resistance management planning [47]. Changes in the prevalence of insecticide resistance over time and differences among sibling species are apparent in the dataset presented here but a full analysis of this data must take account of the strong spatial bias in sampled locations and potential confounding factors such as variation in the protocols used. The one exception is susceptibility to organophosphates in *An. funestus*, which has remained constant over time at all locations, in agreement with earlier reviews [48]. It is clear that there are far fewer data available for individual species than for complexes [49]. There is, however, sufficient data available for the most important vectors species to allow variation in resistance at the species level to be considered and combined with species distributions.

## Conclusions

Sibling species within the Gambiae complex display important differences in their geographical distributions and the same appears to be true for prevalence of insecticide resistance. The most important malaria vector sibling species in Africa have been mapped here for the first time and the evidence for insecticide resistance in these species has been summarised. The species-specific distributions can now be coupled with data on insecticide resistance, behaviour and vector status to make better informed decisions on vector control policy.

## Additional files

**Additional file 1.** Environmental covariates used in the model.

**Additional file 2.** Presence and background datasets for each species. Each dataset is provided in a separate worksheet within the workbook.

**Additional file 3.** Maps showing the spatial distribution of the data that went into the model.

**Additional file 4.** Additional maps for the Gambiae complex, Funestus subgroup, and Funestus group.

**Additional file 5.** Tables of AUC values and top predictors, and maps showing the prediction ranges.

**Additional file 6.** Geotiff that can be opened in GIS software such as QGIS (http://www.qgis.org/) or ArcMap (http://www.esri.com/software/arcgis). Model output data for *An. arabiensis*. Band 1 contains the mean values per pixel, band 2 contains the median values, band 3 contains the 2.5% quantile values, and band 4 contains the 97.5% quantile values.

**Additional file 7.** geotiff that can be opened in GIS software such as QGIS (http://www.qgis.org/) or ArcMap (http://www.esri.com/software/arcgis). Model output data for *An. coluzzii.* Band 1 contains the mean values per pixel, band 2 contains the median values, band 3 contains the 2.5% quantile values, and band 4 contains the 97.5% quantile values.

**Additional file 8.** geotiff that can be opened in GIS software such as QGIS (http://www.qgis.org/) or ArcMap (http://www.esri.com/software/arcgis). Model output data for *An. funestus.* Band 1 contains the mean values per pixel, band 2 contains the median values, band 3 contains the 2.5% quantile values, and band 4 contains the 97.5% quantile values.

**Additional file 9.** geotiff that can be opened in GIS software such as QGIS (http://www.qgis.org/) or ArcMap (http://www.esri.com/software/arcgis). Model output data for *An. gambiae.* Band 1 contains the mean values per pixel, band 2 contains the median values, band 3 contains the 2.5% quantile values, and band 4 contains the 97.5% quantile values.

**Additional file 10.** geotiff that can be opened in GIS software such as QGIS (http://www.qgis.org/) or ArcMap (http://www.esri.com/software/arcgis). Model output data for *An. melas.* Band 1 contains the mean values per pixel, band 2 contains the median values, band 3 contains the 2.5% quantile values, and band 4 contains the 97.5% quantile values.

**Additional file 11.** geotiff that can be opened in GIS software such as QGIS (http://www.qgis.org/) or ArcMap (http://www.esri.com/software/arcgis). Model output data for *An. merus.* Band 1 contains the mean values per pixel, band 2 contains the median values, band 3 contains the 2.5% quantile values, and band 4 contains the 97.5% quantile values.

**Additional file 12.** geotiff that can be opened in GIS software such as QGIS (http://www.qgis.org/) or ArcMap (http://www.esri.com/software/arcgis). Model output data for the Gambiae complex. Band 1 contains the mean values per pixel, band 2 contains the median values, band 3 contains the 2.5% quantile values, and band 4 contains the 97.5% quantile values.

**Additional file 13.** geotiff that can be opened in GIS software such as QGIS (http://www.qgis.org/) or ArcMap (http://www.esri.com/software/arcgis). Model output data for the Funestus subgroup. Band 1 contains the mean values per pixel, band 2 contains the median values, band 3 contains the 2.5% quantile values, and band 4 contains the 97.5% quantile values.

**Additional file 14.** geotiff that can be opened in GIS software such as QGIS (http://www.qgis.org/) or ArcMap (http://www.esri.com/software/arcgis). Model output data for the Funestus group. Band 1 contains the mean values per pixel, band 2 contains the median values, band 3 contains the 2.5% quantile values, and band 4 contains the 97.5% quantile values.

**Additional file 15.** Tables of summary insecticide resistance data. A separate worksheet is provided for each insecticide class for each of the Gambiae complex and the Funestus group.

## References

[1] Battle KE, Gething PW, Elyazar IRF, Moyes CL, Sinka ME, Howes RE (2012). The global public health significance of *Plasmodium vivax*. Adv Parasitol.

[2] Sinka ME, Bangs MJ, Manguin S, Rubio-Palis Y, Chareonviriyaphap T, Coetzee M (2012). A global map of dominant malaria vectors. Parasit Vectors.

[3] Coetzee M, Hunt RH, Wilkerson R, Della Torre A, Coulibaly MB, Besansky NJ (2013). *Anopheles coluzzii* and *Anopheles amharicus*, new members of the *Anopheles gambiae* complex. Zootaxa.

[4] Kipyab PC, Khaemba BM, Mwangangi JM, Mbogo CM (2013). The bionomics of *Anopheles merus* (Diptera: Culicidae) along the Kenyan coast. Parasit Vectors.

[5] Ebenezer A, Noutcha AEM, Okiwelu SN (2016). Relationship of annual entomological inoculation rates to malaria transmission indices, Bayelsa State, Nigeria. J Vector Borne Dis..

[6] Sinka ME, Bangs MJ, Manguin S, Coetzee M, Mbogo CM, Hemingway J (2010). The dominant *Anopheles* vectors of human malaria in Africa, Europe and the Middle East: occurrence data, distribution maps and bionomic precis. Parasit Vectors.

[7] Hemingway J, Ranson H, Magill A, Kolaczinski J, Fornadel C, Gimnig J (2016). Averting a malaria disaster: will insecticide resistance derail malaria control?. Lancet.

[8] Massey NC, Garrod G, Wiebe A, Henry AJ, Huang Z, Moyes CL (2016). A global bionomic database for the dominant vectors of human malaria. Sci Data.

[9] Moyes CL, Temperley WH, Henry AJ, Burgert CR, Hay SI (2013). Providing open access data online to advance malaria research and control. Malar J.

[10] Phillips SJ, Dudik M, Elith J, Graham CH, Lehmann A, Leathwick J (2009). Sample selection bias and presence-only distribution models: implications for background and pseudo-absence data. Ecol Appl.

[11] United Nations Environmental Programme. Biomes of Africa. In Africa Atlas of Our Changing Environment. Johannesburg; 2008.

[12] Dia I, Guelbeogo MW, Ayala D. Advances and perspectives in the study of the malaria mosquito *Anopheles funestus*. In: Manguin S, editor. Anopheles mosquitoes—new insights into malaria vectors. Rijeka: In Tech Publisher; 2013. p. 197–220.

[13] della Torre A, Tu ZJ, Petrarca V (2005). On the distribution and genetic differentiation of *Anopheles gambiae* s.s. molecular forms. Insect Biochem Mol Biol.

[14] Weiss DJ, Mappin B, Dalrymple U, Bhatt S, Cameron E, Hay SI (2015). Re-examining environmental correlates of *Plasmodium falciparum* malaria endemicity: a data-intensive variable selection approach. Malar J.

[15] De’ath G (2007). Boosted trees for ecological modeling and prediction. Ecology.

[16] Elith J, Graham CH, Anderson RP, Dudik M, Ferrier S, Guisan A (2006). Novel methods improve prediction of species’ distributions from occurrence data. Ecography.

[17] Elith J, Leathwick JR, Hastie T (2008). A working guide to boosted regression trees. J Anim Ecol.

[18] Moyes CL, Shearer FM, Huang Z, Wiebe A, Gibson HS, Nijman V (2016). Predicting the geographical distributions of the macaque hosts and mosquito vectors of *Plasmodium knowlesi* malaria in forested and non-forested areas. Parasit Vectors.

[19] Shearer FM, Huang Z, Weiss DJ, Wiebe A, Gibson HS, Battle KE (2016). Estimating geographical variation in the risk of zoonotic *Plasmodium knowlesi* infection in countries eliminating malaria. PLoS Negl Trop Dis.

[20] Fleiss J, Levin B, Paik M (2003). Statistical methods for rates and proportions.

[21] Drake JM, Beier JC (2014). Ecological niche and potential distribution of *Anopheles arabiensis* in Africa in 2050. Malar J.

[22] Fossog BT, Ayala D, Acevedo P, Kengne P, Mebuy INA, Makanga B (2015). Habitat segregation and ecological character displacement in cryptic African malaria mosquitoes. Evol Appl.

[23] Adamou A, Dao A, Timbine S, Kassogue Y, Yaro AS, Diallo M (2011). The contribution of aestivating mosquitoes to the persistence of *Anopheles gambiae* in the Sahel. Malar J.

[24] Lee Y, Meneses CR, Fofana A, Lanzaro GC (2009). Desiccation resistance among subpopulations of *Anopheles gambiae* s.s. from Selinkenyi, Mali. J Med Entomol.

[25] Lyons CL, Coetzee M, Chown SL (2013). Stable and fluctuating temperature effects on the development rate and survival of two malaria vectors, *Anopheles arabiensis* and *Anopheles funestus*. Parasit Vectors.

[26] de Souza D, Kelly-Hope L, Lawson B, Wilson M, Boakye D (2010). Environmental factors associated with the distribution of *Anopheles gambiae* s.s in Ghana; an important vector of lymphatic filariasis and malaria. PLoS ONE.

[27] Huestis DL, Yaro AS, Traore AI, Adamou A, Kassogue Y, Diallo M (2011). Variation in metabolic rate of *Anopheles gambiae* and *A. arabiensis* in a Sahelian village. J Exp Biol.

[28] Kelly-Hope LA, Hemingway J, McKenzie FE (2009). Environmental factors associated with the malaria vectors *Anopheles gambiae* and *Anopheles funestus* in Kenya. Malar J.

[29] Kipyab PC, Khaemba BM, Mwangangi JM, Mbogo CM (2015). The physicochemical and environmental factors affecting the distribution of *Anopheles merus* along the Kenyan coast. Parasit Vectors.

[30] Kirby MJ, Lindsay SW (2009). Effect of temperature and inter-specific competition on the development and survival of *Anopheles gambiae* sensu stricto and *An. arabiensis* larvae. Acta Trop.

[31] Lyons CL, Coetzee M, Terblanche JS, Chown SL (2012). Thermal limits of wild and laboratory strains of two African malaria vector species, *Anopheles arabiensis* and *Anopheles funestus*. Malar J.

[32] Paaijmans KP, Huijben S, Githeko AK, Takken W (2009). Competitive interactions between larvae of the malaria mosquitoes *Anopheles arabiensis* and *Anopheles gambiae* under semi-field conditions in western Kenya. Acta Trop.

[33] Afrane YA, Zhou G, Lawson BW, Githeko AK, Yan G (2007). Life-table analysis of *Anopheles arabiensis* in western Kenya highlands: effects of land covers on larval and adult survivorship. Am J Trop Med Hyg.

[34] Minakawa N, Dida GO, Sonye GO, Futami K, Njenga SM (2012). Malaria vectors in Lake Victoria and adjacent habitats in Western Kenya. PLoS ONE.

[35] Dery DB, Brown C, Asante KP, Adams M, Dosoo D, Amenga-Etego S (2010). Patterns and seasonality of malaria transmission in the forest-savannah transitional zones of Ghana. Malar J.

[36] Guelbeogo WM, Sagnon N, Grushko O, Yameogo MA, Boccolini D, Besansky NJ (2009). Seasonal distribution of *Anopheles funestus* chromosomal forms from Burkina Faso. Malar J.

[37] Mwangangi JM, Mbogo CM, Orindi BO, Muturi EJ, Midega JT, Nzovu J (2013). Shifts in malaria vector species composition and transmission dynamics along the Kenyan coast over the past 20 years. Malar J.

[38] Simard F, Lehmann T, Lemasson JJ, Diatta M, Fontenille D (2000). Persistence of *Anopheles arabiensis* during the severe dry season conditions in Senegal: an indirect approach using microsatellite loci. Insect Molec Biol.

[39] Athrey G, Hodges TK, Reddy MR, Overgaard HJ, Matias A, Ridl FC (2012). The effective population size of malaria mosquitoes: large impact of vector control. PLoS Genet.

[40] Bhatt S, Weiss DJ, Cameron E, Bisanzio D, Mappin B, Dalrymple U (2015). The effect of malaria control on *Plasmodium falciparum* in Africa between 2000 and 2015. Nature.

[41] Crook SE, Baptista A (1995). The effect of permethrin-impregnated wall-curtains on malaria transmission and morbidity in the suburbs of Maputo, Mozambique. Trop Geogr Med.

[42] Gimnig JE, Vulule JM, Lo TQ, Kamau L, Kolczak MS, Phillips-Howard PA (2003). Impact of permethrin-treated bed nets on entomologic indices in an area of intense year-round malaria transmission. Am J Trop Med Hyg.

[43] Govella NJ, Chaki PP, Killeen GF (2013). Entomological surveillance of behavioural resilience and resistance in residual malaria vector populations. Malar J.

[44] Sinka ME, Golding N, Massey NC, Wiebe A, Huang Z, Hay SI (2016). Modelling the relative abundance of the primary African vectors of malaria before and after the implementation of indoor, insecticide-based vector control. Malar J.

[45] Killeen GF, Kiware SS, Okumu FO, Sinka ME, Moyes CL, Massey NC, et al. Going beyond personal protection against mosquito bites to eliminate malaria transmission: population suppression of malaria vectors that exploit both human and animal blood. BMJ Glob Health. http://archive.lstmed.ac.uk/6378/ (in press). 10.1136/bmjgh-2016-000198 PMC5444054 28589015

[46] Killeen GF (2014). Characterizing, controlling and eliminating residual malaria transmission. Malar J.

[47] Chanda E, Thomsen EK, Musapa M, Kamuliwo M, Brogdon WG, Norris DE (2016). An operational framework for insecticide resistance management planning. Emerg Infect Dis.

[48] Coetzee M, Koekemoer LL (2013). Molecular systematics and insecticide resistance in the major African malaria vector *Anopheles funestus*. An Rev Entomol.

[49] Coleman M, Hemingway J, Gleave K, Wiebe A, Gething PW, Moyes CL. Developing global maps of insecticide resistance risk to improve vector control. Malar J. doi:10.1186/s12936-017-1733-z. 10.1186/s12936-017-1733-z PMC5320685 28222727

[50] SEEG-Oxford SDM code at the GitHub repository. https://github.com/SEEG-Oxford/seegSDM.

