# Supplementary material for: Geographical distributions of African malaria vector sibling species and evidence for insecticide resistance
Source: Malar J. 2017 Feb 20;16:85. doi: 10.1186/s12936-017-1734-y (PMC5319841; doi:10.1186/s12936-017-1734-y)
Supplement: Supplementary file 1 — Additional file 1. Environmental covariates used in the model. [file 12936_2017_1734_MOESM1_ESM.docx]

**Additional file 1**: **Environmental covariates included in the species distribution models**

| **Name** | **Description** | **Source** |
| --- | --- | --- |
| mean enhanced vegetation index | The enhanced vegetation index is linked to vegetation cover density and is related to precipitation. It is measured on a scale from 0 (low) to 1 (high). The corrected mean value across each month of the year, for the period 2000 to 2014, was used. | MODISSatelliteImagery [1] |
| seasonality in the enhanced vegetation index | The standard deviation in 8-daily values from 2000 to 2014, representing seasonality in the index, was used. |  |
| mean daytime temperature | Daytime land surface temperature in degrees Celsius. The corrected mean value across each month of the year, for the period 2000 to 2014. |  |
| seasonality in daytime temperature | The the standard deviation in 8-daily values from 2000 to 2014, representing seasonality in temperature, was used. |  |
| mean nighttime temperature | Nighttime land surface temperature in degrees Celsius. The corrected mean value across each month of the year, for the period 2000 to 2014, was used. |  |
| seasonality in nighttime temperature | The standard deviation in 8-daily values from 2000 to 2014, representing seasonality in temperature, was used. |  |
| mean tasselled cap brightness | Tasselled cap brightness (TCB) is associated with moisture in areas with bare soils or senescent vegetation. The corrected mean value across each month of the year, for the period 2000 to 2014, was used. |  |
| seasonality in tasselled cap brightness | The standard deviation in 8-daily values from 2000 to 2014, representing seasonality in TCB, was used. |  |
| mean tasselled cap wetness | Tasselled cap wetness is associated with surface moisture. The corrected mean value across each month of the year, for the period 2000 to 2014, was used. |  |
| seasonality in tasselled cap wetness | The standard deviation in 8-daily values from 2000 to 2014, representing seasonality in TCW, was used. |  |
| human population density | Human population density in 2010. | WorldPop &  Gridded Population of the World [2, 3] |
| elevation | Elevation in metres. | Shuttle Radar Topography Mission [4] |

**Table S1**. Synoptic covariates that provide values for every 5 x 5 km pixel, across a number of years.

| **Temporal Covariates** | **Description** | **Source** |
| --- | --- | --- |
| closed shrubland cover | Proportional cover of land with woody vegetation <2m tall and cover >60%, annually from 2001 to 2012. | International Geosphere and Biosphere Programme (IGBP) land cover classification within MODIS dataset [5] |
| cropland/natural vegetation mosaic cover | Proportional cover of land with a mosaic of croplands, forest, shrublands and grasslands in which no one component comprises >60%, annually from 2001 to 2012. |  |
| cropland cover | Proportional cover of land covered with temporary crops followed by harvest and bare soil, annually from 2001 to 2012. |  |
| grassland cover | Proportional cover of land with herbaceous vegetation, and tree/shrub cover <10%, annually from 2001 to 2012. |  |
| open shrubland cover | Proportional cover of land with woody vegetation <2m tall and cover 10-60%, annually from 2001 to 2012. |  |
| permanent wetlands | Proportional cover of land with a permanent mixture of water and herbaceous or woody vegetation over extensive areas, annually from 2001 to 2012. |  |
| savannah cover | Proportional cover of land with canopy cover 10-30% and height >2m, with understory vegetation, annually from 2001 to 2012. |  |
| urban and built-up cover | Proportional cover of land covered by buildings and other man-made structures, annually from 2001 to 2012. |  |
| woody savannah cover | Proportional cover of land with canopy cover 30-60% and height >2m, with understory vegetation, annually from 2001 to 2012. |  |
| deciduous broadleaf forest cover | Proportional cover of lands with deciduous broadleaf canopy cover >60% and height >2m, annually from 2001 to 2012. |  |
| deciduous needleleaf forest cover | Proportional cover of lands with deciduous needleleaf canopy cover >60% and height >2m, annually from 2001 to 2012. |  |
| evergreen broadleaf forest cover | Proportional cover of lands with evergreen broadleaf canopy cover >60% and height >2m, annually from 2001 to 2012. |  |
| evergreen needleleaf forest cover | Proportional cover of lands with evergreen needleleaf canopy cover >60% and height >2m, annually from 2001 to 2012. |  |
| mixed forest cover | Proportional cover of land with mixed forest canopy cover >60% and height >2m, annually from 2001 to 2012. |  |

**Table S2**. Annual covariates that provide values for every 5 x 5 km pixel, for each year.

1. MODIS online data repository. 2014. http://e4ftl01.cr.usgs.gov/MODIS_Composites/MOTA/MCD12Q1.051/2001.01.01/. Accessed 20 Apr 2014.
2. WorldPop population data repository. 2015. http://www.worldpop.org.uk/data/get_data/. Accessed 06 Jul 2015.
3. Gridded Population of the World spatially diaggregated populations layers. 2015. http://sedac.ciesin.columbia.edu/data/collection/gpw-v3/sets/browse. Accessed 06 Jul 2015.
4. Farr TG, Rosen PA, Caro E, Crippen R, Duren R, Hensley S: **The Shuttle Radar Topography Mission**. *Reviews of Geophysics* 2007, **45**:RG2004.
5. Friedl MA, Sulla-Menashe D, Tan B, Schneider A, Ramankutty N, Sibley A, et al: MODIS Collection 5 global land cover: algorithm refinements and characterization of new datasets. *Remote Sensing of Environment* 2010, **114:**1:168-82.
