# Supplementary material for: Geographical distributions of African malaria vector sibling species and evidence for insecticide resistance
Source: Malar J. 2017 Feb 20;16:85. doi: 10.1186/s12936-017-1734-y (PMC5319841; doi:10.1186/s12936-017-1734-y)

**Additional file 3: Data maps**

The presence and background data that went into each model is shown. The background data is divided into two classes; class one are locations from surveys that used molecular methods that would have detected the species being modelled, and class two did not. (a,b) *An. coluzzii*. (c,d) *An. gambiae* (e,f) *An. arabiensis*. (g,h) *An. funestus.* (i,j) An*. melas*. (k,l) *An. merus*.


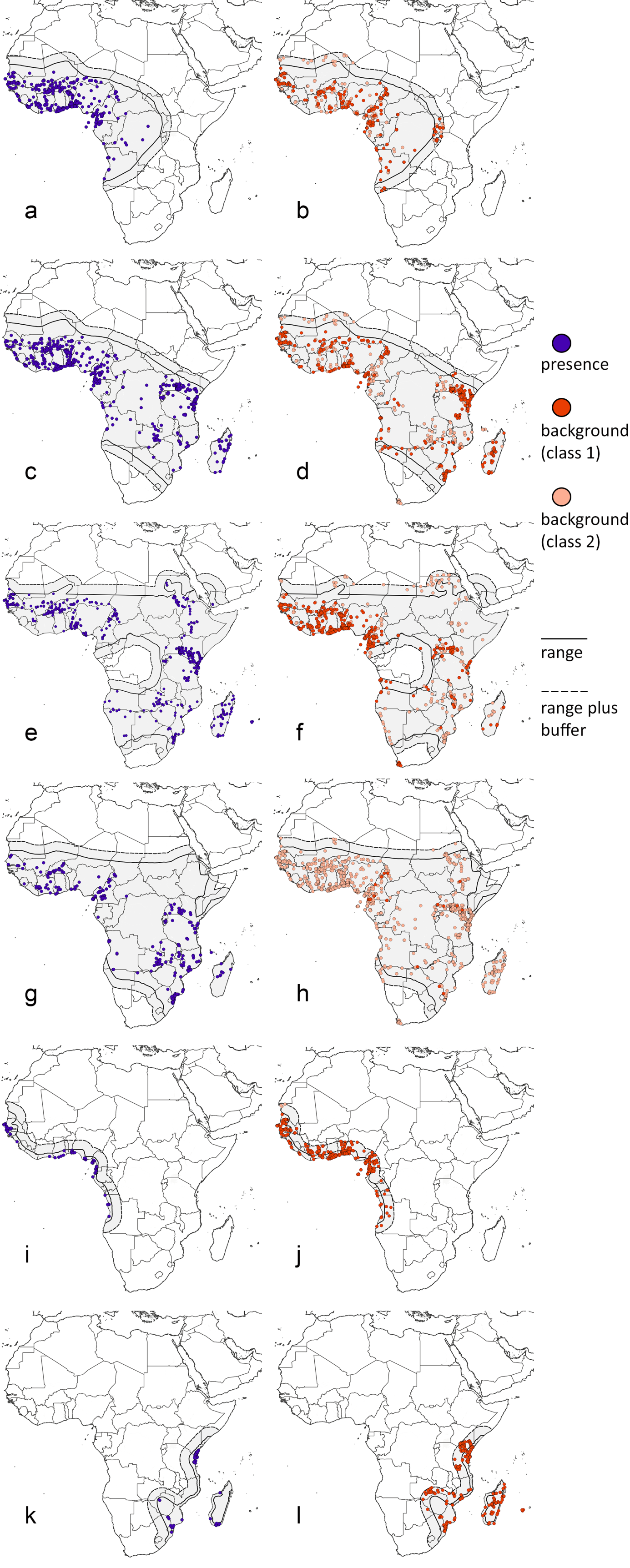


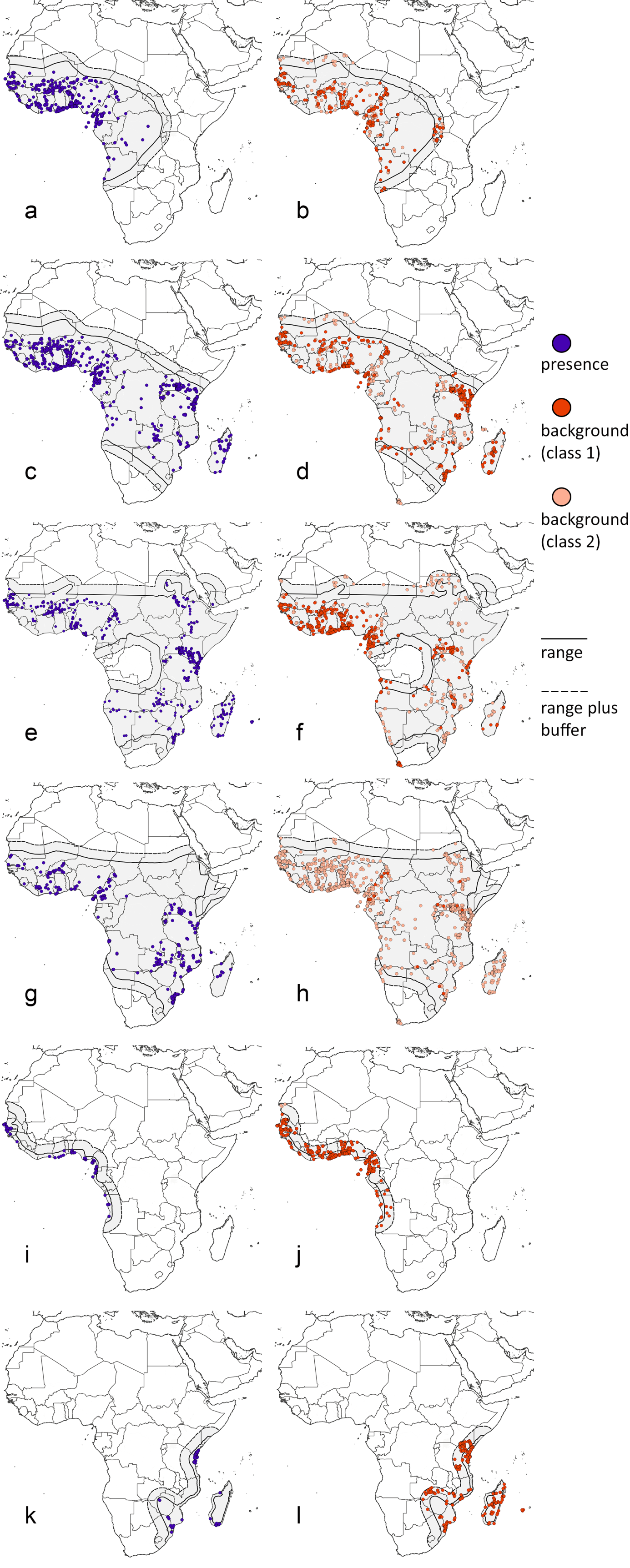

Supplement: Supplementary file 3 — Additional file 3. Maps showing the spatial distribution of the data that went into the model. [file 12936_2017_1734_MOESM3_ESM.docx]
