# Supplementary material for: Geographical distributions of African malaria vector sibling species and evidence for insecticide resistance
Source: Malar J. 2017 Feb 20;16:85. doi: 10.1186/s12936-017-1734-y (PMC5319841; doi:10.1186/s12936-017-1734-y)
Supplement: Supplementary file 4 — Additional file 4. Additional maps for the Gambiae complex, Funestus subgroup, and Funestus group. [file 12936_2017_1734_MOESM4_ESM.docx]

**Additional file 3: Complex, Subgroup and Group maps**

**
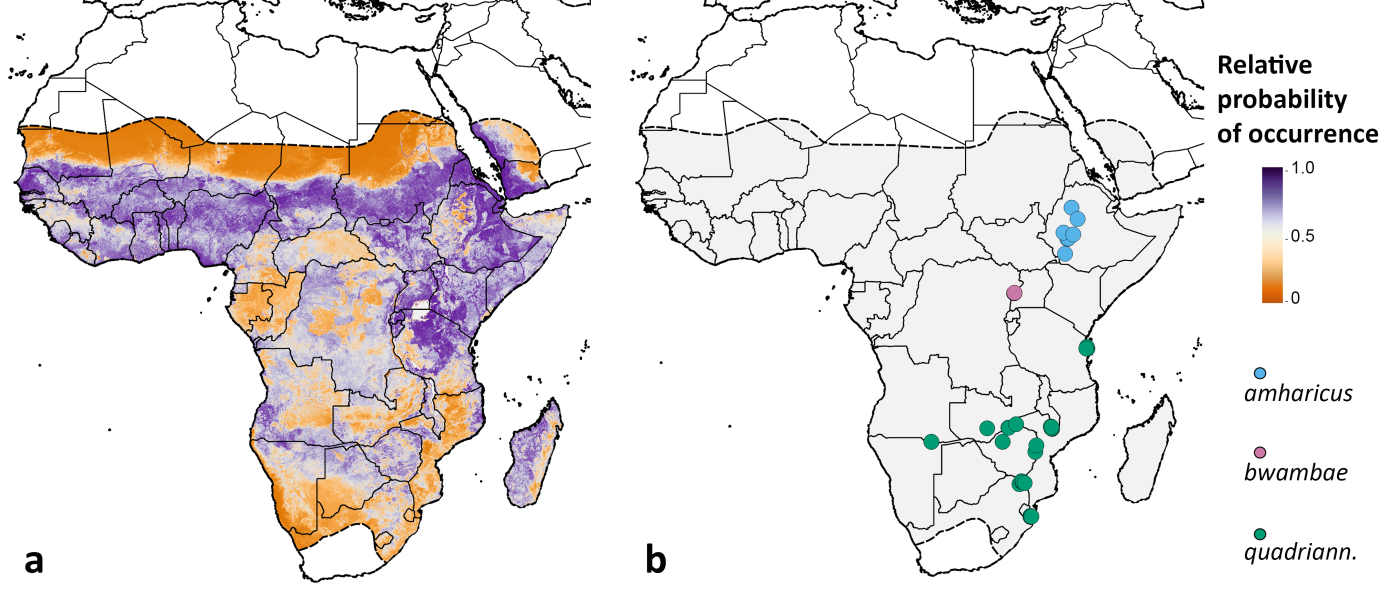
**

**Figure S1.** (a) The predicted relative probability of one or more species from the Gambiae complex occurring, within the complex range plus a 300 km buffer. (b) Species that weren’t modelled individually are shown within the complex range; *Anopheles* *amharicus* (*amharicus*), *An*. *bwambae* (*bwambae*) and *An*. *quadriannulatus* (*quadriann*.).


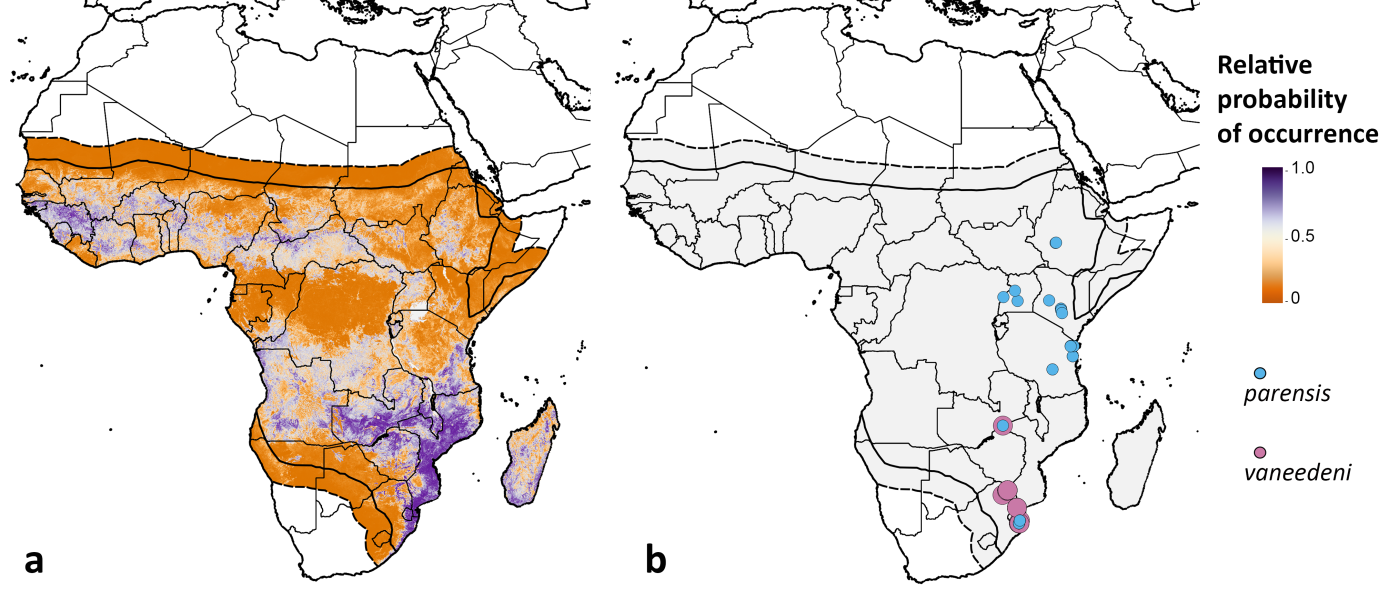


**Figure S2.** (a) The predicted relative probability of one or more species from the Funestus subgroup occurring, within the subgroup range plus a 300 km buffer. (b) Species that weren’t modelled individually are shown within the subgroup range.


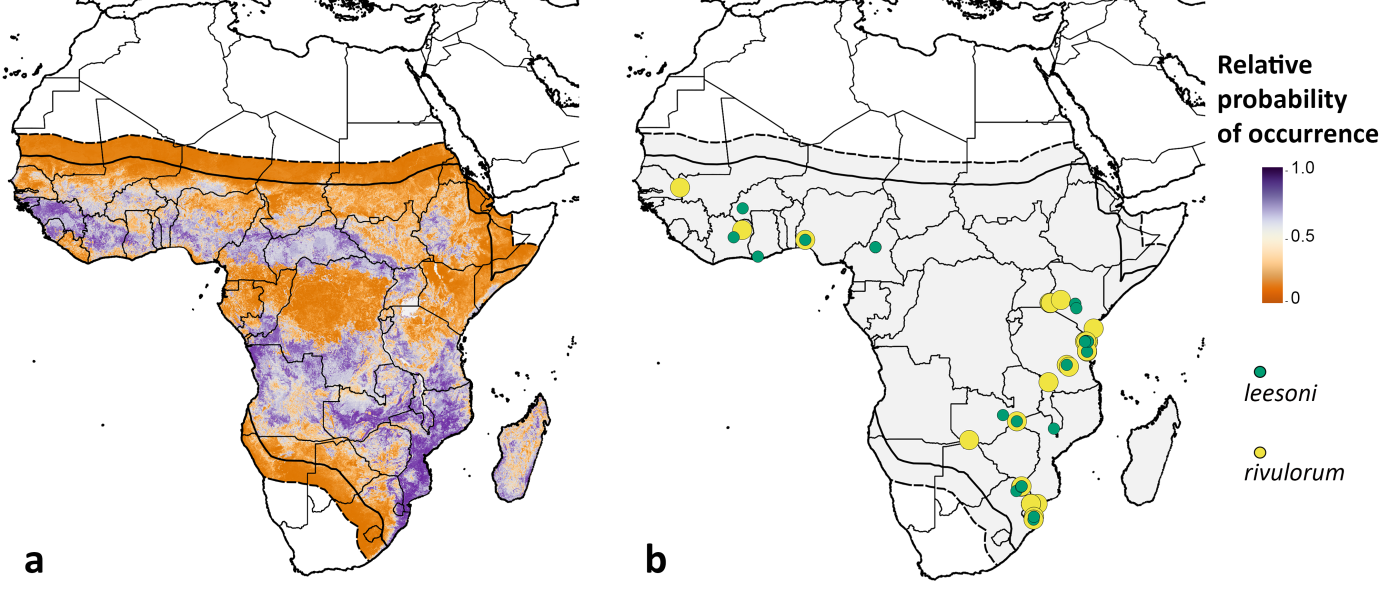


**Figure S3.** (a) The predicted relative probability of one or more species from the Funestus group occurring, within the group range plus a 300 km buffer. (b) Species that weren’t modelled individually are shown within the group range.
