# Supplementary material for: Geographical distributions of African malaria vector sibling species and evidence for insecticide resistance
Source: Malar J. 2017 Feb 20;16:85. doi: 10.1186/s12936-017-1734-y (PMC5319841; doi:10.1186/s12936-017-1734-y)

**Additional File 4 – model validation and top predictors**

The final model output, the mean map, was validated by calculating an AUC value using the withheld dataset of presence and background data points, for each species modelled. The same withheld datasets were used to generate AUC values for previously published predictions. Both sets of AUC values are given in Table S3.

**Table S3**. **Area under the receiver operator curve (AUC) values for each model output.**

|  | **AUC value** | |
| --- | --- | --- |
| **Species, complex or group** | **Model outputs from this study** | **Previously published model outputs^1^** |
| *An. arabiensis* | 0.870 | 0.704 |
| *An. coluzzii* | 0.783 |  |
| *An. gambiae* | 0.778 |  |
| *An. melas* | 0.866 | 0.548 |
| *An. merus* | 0.804 | 0.536 |
| Gambiae complex | 0.757 |  |
| *An. funestus* | 0.824 |  |
| Funestus subgroup | 0.806 | 0.626 |
| Funestus group | 0.796 |  |

1. Sinka ME, Bangs MJ, Manguin S, Coetzee M, Mbogo CM, Hemingway J, Patil AP, Temperley WH, Gething PW, Kabaria CW, et al: **The dominant Anopheles vectors of human malaria in Africa, Europe and the Middle East: occurrence data, distribution maps and bionomic precis.** *Parasites & Vectors* 2010, **3**.

In addition, an AUC value was calculated for each submodel as part of the modelling process, given as the mean of the cross-validated AUC across ten folds. A ten-fold cross validation was used as the default but five-fold cross validation was used for *An. merus* and *An. melas* due to their smaller data numbers. The cross-validation process separates the dataset into ten subsets containing approximately the same number of presence and background points. The submodel is then iteratively trained using nine subsets of the data, and the performance in predicting the withheld data is evaluated by generating AUC values. During cross-validation, we used a pairwise distance sampling procedure to prevent inflation of evaluation statistics due to spatial sorting bias in the cross-validation subsets^2^. This pairwise distribution sampling procedure avoids inflating this AUC generated for the submodels. The AUCs were then averaged across the 200 submodels and the mean AUC values generated by the ensemble are given in Table S4.

1. Hijmans RJ: **Cross-validation of species distribution models: removing spatial sorting bias and calibration with a null model**. *Ecology* 2012, **93**:679-688.

**Table S4. Mean AUC values generated by the cross-validation performed by each ensemble model.**

| **Species, complex or group** | **Mean AUC value** |
| --- | --- |
| *An. arabiensis* | 0.866 |
| *An. coluzzii* | 0.823 |
| *An. gambiae* | 0.796 |
| *An. melas* | 0.710 |
| *An. merus* | 0.694 |
| Gambiae complex | 0.878 |
| *An. funestus* | 0.709 |
| Funestus subgroup | 0.719 |
| Funestus group | 0.699 |

**Tables S5**. **The covariates with most influence on each model.** The most influential covariate is ranked first. The table is provided in two parts and abbreviations are defined below.

| **Species** | ***arabiensis*** | ***coluzzii*** | ***gambiae*** | ***melas*** | ***merus*** |
| --- | --- | --- | --- | --- | --- |
| **Rank** |  |  |  |  |  |
| 1 | seasonality in wetness (TCW) | night temperature | seasonality in night temp. | elevation | elevation |
| 2 | elevation | seasonality in wetness (TCB) | night temperature | seasonality in night temp. | seasonality in night temp. |
| 3 | day temperature | elevation | elevation | night temperature | seasonality in greenness (EVI) |
| 4 | seasonality in greenness (EVI) | seasonality in day temp. | cropland/natural veg. mosaic cover | human pop. density | woody savannah cover |
| 5 | seasonality in night temp. | seasonality in wetness (TCW) | human pop. density | permanent wetland cover | night temperature |
| 6 | human pop. density | seasonality in night temp. | greenness (EVI) | wetness (TCB) | greenness (EVI) |
| 7 | night temperature | wetness (TCB) | seasonality in wetness (TCB) | day temperature | seasonality in wetness (TCB) |
| 8 | seasonality in day temp. | cropland/natural veg. mosaic cover | seasonality in greenness (EVI) |  |  |
| 9 | seasonality in wetness (TCB) | human pop. density | seasonality in day temp. |  |  |
| 10 |  | seasonality in greenness (EVI) | day temperature |  |  |
| 11 |  |  | wetness (TCW) |  |  |
| 12 |  |  | seasonality in wetness (TCW) |  |  |

| **Species** | ***funestus*** | **Funestus subgroup** | **Funestus group** |  | **Gambiae complex** |
| --- | --- | --- | --- | --- | --- |
| **Rank** |  |  |  |  |  |
| 1 | woody savannah cover | e/green b/leaf forest cover | woody savannah cover |  | seasonality in night temp. |
| 2 | seasonality in night temp. | savannah cover | seasonality in wetness (TCW) |  | seasonality in day temp. |
| 3 | seasonality in wetness (TCW) | woody savannah cover | e/green b/leaf forest cover |  | night temperature |
| 4 | day temperature | day temperature | greenness (EVI) |  | human pop. density |
| 5 | seasonality in wetness (TCB) | greenness (EVI) | night temperature |  | day temperature |
| 6 | savannah cover | seasonality in wetness (TCW) | day temperature |  |  |
| 7 | human pop. density | seasonality in night temp. | savannah cover |  |  |
| 8 | wetness (TCB) | seasonality in greenness (EVI) | seasonality in day temp. |  |  |
| 9 | seasonality in day temp. | night temperature | seasonality in greenness (EVI) |  |  |
| 10 | night temperature | wetness (TCB) | seasonality in wetness (TCB) |  |  |
| 11 | wetness (TCW) |  |  |  |  |

**Abbreviations**

e/green b/leaf= evergreen broadleaf

EVI = enhanced vegetation index

TCB = tasselled cap brightness (a measure of surface moisture on bare areas)

TCW = tasselled cap wetness (a measure of surface moisture)

temp. = temperature

pop. = population

**Maps showing the range between the 2.5th and the 97.5th centile of the model predictions.** (a) *An. coluzzii*. (b) *An. gambiae* (c) *An. arabiensis*. (d) *An. funestus.* (e) An*. melas*. (f) *An. merus*.


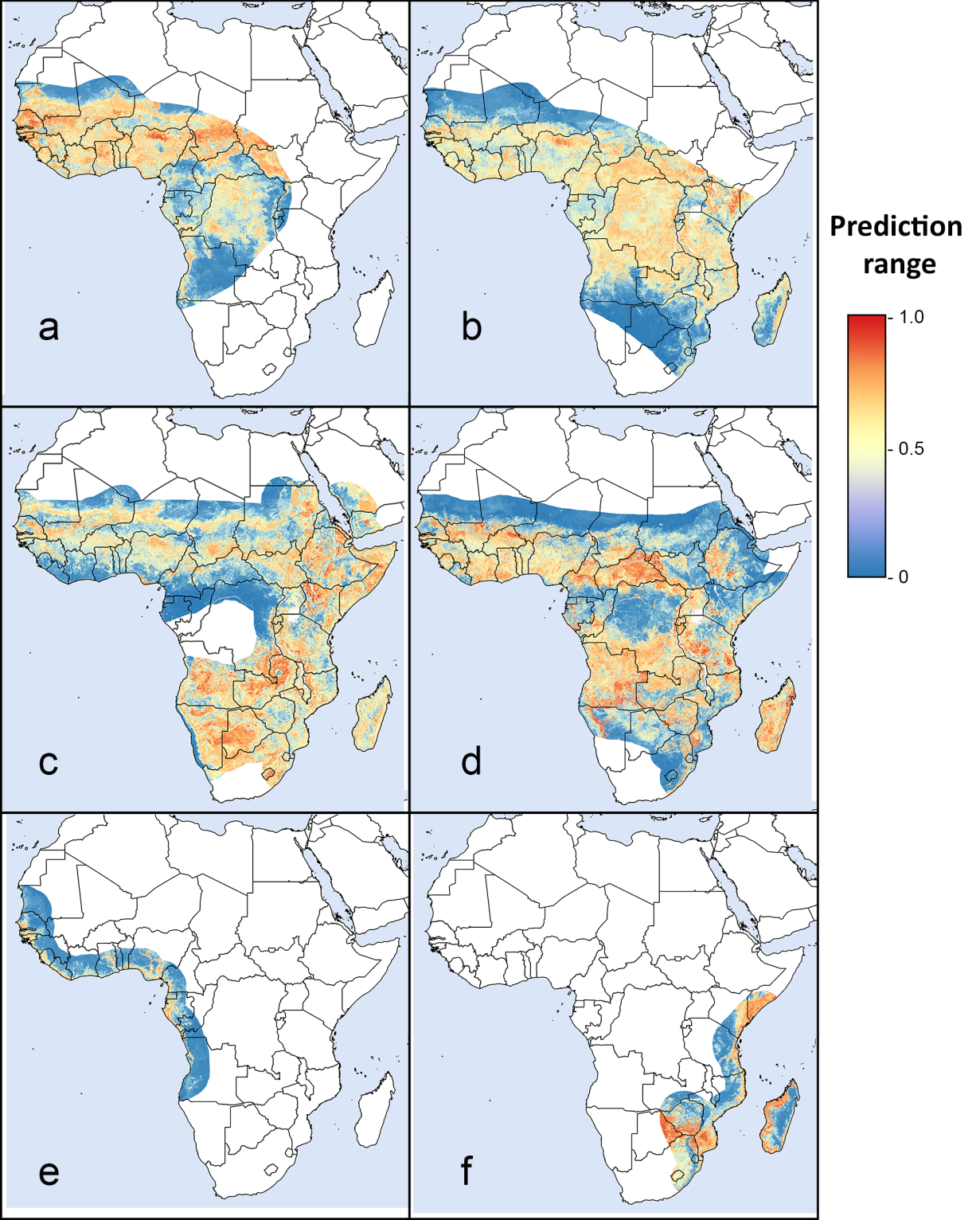

Supplement: Supplementary file 5 — Additional file 5. Tables of AUC values and top predictors, and maps showing the prediction ranges. [file 12936_2017_1734_MOESM5_ESM.docx]
